# Endocrine and inflammatory profiles in type 2 diabetic patients with and without major depressive disorder

**DOI:** 10.1186/1756-0500-6-61

**Published:** 2013-02-14

**Authors:** Adriana Alvarez, Jose Faccioli, Mónica Guinzbourg, María M Castex, Claudia Bayón, Walter Masson, Ignacio Bluro, Andrea Kozak, Patricia Sorroche, Lina Capurro, Luis Grosembacher, Adrián Proietti, Carlos Finkelsztein, Lucas Costa, Patricia Fainstein Day, Arturo Cagide, León E Litwak, Sherita H Golden

**Affiliations:** 1 Diabetes Division, Endocrinology and Nuclear Medicine Department, “Hospital Italiano de Buenos Aires”, Buenos Aires, Argentina; 2 Cardiology Department, “Hospital Italiano de Buenos Aires”, Buenos Aires, Argentina; 3 LBAL (Laboratory for Biological & Artificial Learning) of “Hospital Italiano de Buenos Aires”, Buenos Aires, Argentina; 4 Department of Medicine, Johns Hopkins University School of Medicine and Department of Epidemiology, Johns Hopkins University Bloomberg School of Public Health, Baltimore, USA; 5 Asamblea 1458, 7th floor, Buenos Aires, Argentina

**Keywords:** Depression, Type 2 diabetes mellitus, Cardiovascular disease

## Abstract

**Background:**

There is a high prevalence of depression in individuals with type 2 diabetes mellitus. Depressive disorders are associated with increased medical morbidity and mortality in individuals with diabetes. It has been demonstrated that there is a higher prevalence of diabetic complications among individuals with diabetes and depression compared to those without depression. Several biological alterations have been reported in individuals with depressive disorders, particularly abnormal levels of endocrine-inflammatory markers.

This study aims to determine the prevalence of major depressive disorder (MDD) in type 2 diabetes patients, the prevalence of cardiovascular events in individuals with and without MDD and to compare the endocrine-inflammatory profile between groups.

**Methods:**

The study was approved by the “Comité de Etica de Protocolos de Investigación del Departamento de Docencia e Investigación del Hospital Italiano de Buenos Aires” with the number “1262” and included only patients who provided written informed consent. The study was conducted in accordance with the Declaration of Helsinki and the Habeas Data law on protection of personal data (Law Nª 25326, Argentina).

Type 2 diabetes patients (n = 61) were included and they were classified as having MDD or not according to DSM-IV. Macrovascular disease was obtained from the medical history. Additionally, the intima-media thickness of the common carotid, carotid bifurcations and internal carotid arteries was measured non-invasively by two-dimensional ultrasound imaging. Fasting glucose, fasting lipid profile, inflammatory (CRP, TNF-α) and endocrine (urine free cortisol and saliva cortisol) markers. Student t tests were used to compare means for normally distributed variables and Mann-Whitney test for variables without normal distribution. Relative frequencies were calculated and a chi-square analysis was conducted. Data were expressed as mean ± standard deviation (SD) or median and interquartile range. Multivariable logistic regression was used to determine the relative odds of clinical cardiovascular disease in individuals with compared to those without depression. Differences were considered significant using a two-sided p < 0.05.

**Results:**

21 patients (34%) had MDD and 40 patients (66%) didn’t have MDD. Diabetic patients with MDD had significantly higher CRP levels (4.1(1.9-7.6) vs 1.5(0.5-4.4) mg/l; p = 0.02) and 24-hour urine free cortisol (71.4 ± 21.3 vs 59.8 ± 29.3 ug/24 h; p = 0.03). The other metabolic and inflammatory parameters were not statistically different between groups. There was a significantly higher prevalence of cardiovascular events in individuals with MDD: 38% for the depressive group vs 15% for non-depressive group, p = 0.04). Patients with MDD had a 3.5-fold greater odd of having cardiovascular disease.

**Conclusions:**

Diabetic patients with depression are more likely to have cardiovascular events, and different factors can determine this high association.

## Background

Type 2 diabetes is a chronic illness with established cross-sectional and longitudinal relationships with depression. There is a high prevalence of depression in individuals with type 2 diabetes mellitus. A meta-analysis and two systematic reviews [1-3] reported that individuals with diabetes had a 2.9-fold significantly increased odds of having depression compared to non-diabetic individuals. Depressive disorders are associated with increased medical morbidity and mortality in individuals with diabetes [4]. It was demonstrated that there is a higher prevalence of diabetes complications, including retinopathy, nephropathy, neuropathy, macrovascular complications and sexual dysfunction, among individuals with diabetes and depression compared to those without depression [5].

Depression has been associated with poor adherence to diet and exercise regimens and poor glycemic control, including hyperglycemia and high hemoglobin A1c (HbA1c) levels [6-8]. Several studies, recently summarized in a meta-analysis by Mezuk et al [9], have shown that depression is associated with an approximately 60% increased risk of type 2 diabetes. Thus, depression is a modifiable risk factor whose treatment could prevent the development of type 2 diabetes and improve glycemic control and health outcomes in patients with depression and pre-existing diabetes.

Several biological alterations have been reported in individuals with depressive disorders that might increase the risk for type 2 diabetes, including activity of the hypothalamic-pituitary-adrenal (HPA) axis leading to hypercortisolism, sympathetic nervous system (SNS) activation, increases in C-reactive protein (CRP) and inflammatory cytokines as tumor necrosis factor (TNF)-alpha and interleukin (IL)-6, and alterations in norepinephrine and serotonin metabolism [4-8].

The aims of this study were (1) to determine the prevalence of major depression in a sample of individuals with type 2 diabetes using a diagnostic psychiatric interview, (2) to determine the prevalence of cardiovascular risk factors and clinical cardiovascular events in individuals with and without depression and, (3) to characterize the endocrine-inflammatory profile in this population.

## Methods

### Study population

Sixty one patients (30 men and 31 women), aged 18 to 65 years, with physician-diagnosed type 2 diabetes mellitus were included in this observational study. Patients were recruited from the Diabetes Division, Endocrinology and Nuclear Medicine Service of Buenos Aires Italian Hospital, Argentina from 12/05/2008 to 10/12/2009. To be included in the study, patients had to have been followed up in the hospital for at least one year and been able to understand and cooperate with study procedures. Patients were excluded if they were being treated with corticosteroids or rimonabant (because they could produce mood disorders) or had any clinically organic disorder that could interfere with the results. The study was approved by CEPI (Comité de Etica para Protocolos de Investigación del Hospital Italiano de Buenos AIres) a local ethics committee and included only patients who provided written informed consent. The study was conducted in accordance with the Declaration of Helsinki and the Habeas Data law on protection of personal data (Law Nº 25326, Argentina). Each participant was assigned a number by which he/she was identified to keep his or her privacy.

### Psychiatric evaluation

After the evaluation by a psychiatrist and a psychologist from the Mental Health Division of the hospital the patients were classified as having MDD or not according to Diagnostic and Statistical Manual of Mental Disorder criteria (DSM-IV) [10]. The evaluation included an interview with the 17-items Hamilton Rating Scale for Depression (HAM-D17) [11], Mini International Neuropsychiatric Interview (M.I.N.I.) [12] and Beck Depression Inventory (BDI) [13] for depression severity assessment. HAM-D17 scale consists of 17 items, which are rated on a scale from 0 to 2 points or 0 to 4 points depending on severity levels. The total score range is from 0 to 52 points with a remission cut point of 18. The M.I.N.I. is a structured diagnostic interview that explores the psychiatric disorders. The BDI is a multiple-choice self-report questionnaire inventory used for evaluation of subjective consciousness of depressive symptoms and also allows analysis of cognitive versus somatic symptoms of depression. The cut-off value for depression was 24 for the Hamilton scale. Additionally, family history of depression was documented.

### Clinical evaluation

A complete medical history was taken and anthropometric data were collected. Weight and height were measured to calculate body mass index (BMI = weight/ height^2^ [Kg/ m^2^]). Waist circumference (cm) was obtained by measuring the waist in the mean point between the iliac crests and ribs, with the patient standing after expiration. The systolic and diastolic blood pressures (mmHg) were measured with the patient in sitting position after 5 minutes of rest.

### Cardiovascular disease

Macrovascular disease was obtained from the medical clinical history. A history of cardiovascular events was defined as a history of acute myocardial infarction, coronary bypass surgery, angioplasty, angina pectoris, peripheral vascular disease or stroke.

Additionally, the intima-media thickness of the common carotid, carotid bifurcations and internal carotid arteries was measured non-invasively by two-dimensional ultrasound imaging mode, using a Logiq Book XP ultrasound (General Electric®) with a linear transducer of 7.5 MHz in 37 patients (12/21 with depression and 25/40 without depression). Presence of atherosclerotic plaque was defined as: (1) abnormal wall thickness (defined as an intima-media thickness >1.5 mm), (2) abnormal structure (protrusion into the lumen, loss of alignment with the adjacent wall) and (3) abnormal echogenicity of the wall [14]. Increased intima-media thickness was defined as greater than 0.8 mm. An ankle-brachial index was measured according to a standard protocol by trained technicians. Briefly, the participant was asked to lie flat on an examination table, and after 5 minutes of rest, standard arm blood pressure cuffs were applied to the right arm and to each ankle (with the lower end of the bladder within 3 cm of the malleoli). A Doppler vascular probe (7.5 MHz, General Electric) and a standard mercury manometer were used to assess systolic blood pressure in the right brachial artery and in each posterior and anterior tibial artery in rapid succession. The ankle-brachial index in the right and left leg was calculated by dividing the right and the left ankle pressure by the brachial pressure. Of the ankle-brachial index values obtained in each leg, the lower was used in all analysis. A value between 0.9 and 1.3 was considered normal [15].

### Measures

Fasting glucose was measured with the UV-hexokinase method on a Synchron LX20 (Beckman-Coulter) and HbA_1C_ by HPLC on a Variant II instrument (Bio-Rad Laboratories). A fasting lipid profile was also obtained. Cholesterol in serum was measured by an enzymatic method on a Synchron LX 20 (Beckman Coulter). Triglycerides were measured by an enzymatic/GPO-Trinder method on a Synchron LX 20 (Beckman-Coulter). HDL cholesterol was measured by a homogeneous method on the same instrument. The LDL cholesterol concentration was calculated with the Friedewald formula when the serum triglyceride concentration was <400 mg/dL. Apo B was measured by nephelometry on an Immage (Beckman-Coulter). High Sensitivity CRP was measured by a latex turbidimetric method on a Synchron LX20 (Beckman-Coulter). The human TNF-α was measured by an immunoenzymetric assay (Biosource Europe S.A.). Twenty four hour urine free cortisol levels and 23.00 pm saliva cortisol were collected in the outpatient setting and were measured by radioimmunoassay. All the parameters were measured in a centralized laboratory.

### Statistical analysis

The statistical evaluation of data was performed using commercially available SPSS Statistics 17.0 software. Student t tests were applied to compare means for normally distributed variables and Mann-Whitney test for variables without normal distribution. Relative frequencies were calculated and a chi-square analysis was conducted. Data were expressed as mean ± standard deviation (SD) or median and interquartile range. Multivariable logistic regression was used to determine the relative odds of clinical cardiovascular disease in individuals with depression compared to those without depression. Because of the small sample size, only two covariates at a time, determined to be different by depressive status in univariate analyses, were adjusted for. Differences were considered significant using a two-sided p < 0.05.

## Results

### Participant characteristics

Following psychiatric evaluation, 61 participants (mean age 62 ± 7 years) were classified into two groups: 21 patients (34%) with MDD and 40 patients (66%) without depression. The characteristics of individuals with and without depression are summarized in Table 1. There was not significant difference in ratio of male/female patients, age, diabetes duration, insulin therapy, weight, BMI, waist circumference or systolic or diastolic blood pressure between the groups. There was also no difference in family history of depression: depressive group = 5/21 (24%); non-depressive group = 7/40 (18%).

**Table 1:** Participant characteristics by depressive status

	***Depressive group (n = 21)***	***Non-depressive group (n = 40)***	***P value***
**Male/female patients, n (%)**	9 (42.9%) / 12 (57.1%)	21 (52.5%) / 19 (47.5%)	ns
**Age**	63 ± 7.8	62 ± 6.3	ns
**Diabetes duration(years)**	14 ± 10.2	12 ± 6.8	ns
**Insulin treatment**, **n (%)**	12 (57.1%)	19 (47.5%)	ns
**Weight(Kg)**	82.7 ± 22.9	85.9 ± 14.5	ns
**BMI(Kg/m**^**2**^**)**	31.7 ± 6.1	30.8 ± 5.2	ns
**Waist circumference(cm)**	106.9 ± 11.9	105.6 ± 11.4	ns
**Systolic blood pressure(mmHg)**	141.0 ± 13.7	136.4 ± 14.4	ns
**Diastolic blood pressure(mmHg)**	81.4 ± 8.2	80.8 ± 7.1	ns

Data are express as mean ± SD. Student T test or Mann-Whitney test were applied appropriately. Differences were considered significant when p < 0.05.

The prevalence rates of depression in type 2 diabetes in uncontrolled studies are between 17.8 and 39%, with higher much lower rates in controlled studies (between 3.2 and 6.5%). [10] Our study reflects similar rates of the comorbidity than previously published data.

### Metabolic parameters

Several metabolic parameters were assessed and no significant differences were found between groups in glycemic control, lipid profile, ApoB, or TNFα (Table 2). However, compared to those without depression, individuals with depression had significantly higher CRP levels (4.1(1.9-7.6) vs 1.5(0.5-4.4) mg/l; p = 0.02; Mann Whitney test) and 24-hour urine free cortisol (71.4 ± 21.3 vs 59.8 ± 29.3 ug/24 h; p = 0.03; Mann Whitney test).

**Table 2:** Metabolic parameters by depressive status

	***Depressive group (n = 21)***	***Non-depressive group (n = 40)***	***P value***
**Glycemia(RV:70-110 mg/dl)**	161.9 ± 54.9	143.7 ± 38.2	ns
**HbA1c(RV:5-5.8%)**	7.4 ± 1.1	7.5 ± 1.1	ns
**Cholesterol(RV:110-200 mg/dl)**	175.9 ± 34.6	170.0 ± 29.83	ns
**LDL(RV: <100 mg/dl)**	102.6 ± 29.8	102.1 ± 28.6	ns
**HDL(RV: >40 mg/dl)**	46.3 ± 11.0	41.8 ± 9.9	ns
**Triglycerides(RV:50-150 mg/dl)**	133.0 ± 70.3	130.8 ± 65.6	ns
**ApoB(RV:female:51-171 mg/dl;male:56-162 mg/dl)**	89.3 ± 19.6	87.8 ± 18.0	ns
**CRP(RV: <7.48 mg/l)**	4.1(1.9-7.6)	1.5(0.5-4.4)	**0**.**02**
**TNF**-**alpha(RV:4**.**6**-**12.4 pg/ml)**	11.9(8.4-27.4)	11.9(9.1-20.5)	ns
**Urinary free cortisol(RV:100.0 ug/24 h)**	71.4 ± 21.3	59.8 ± 29.3	**0**.**03**
**Saliva cortisol(RV**: **0.7-5.0 nmol/l)**	3.1 ± 1.9	2.2 ± 1.4	ns

Data are express as mean ± SD. CRP and TNF are express as median and interquartile range. Student T test or Mann-Whitney test were applied appropriately. Differences were considered significant when p < 0.05.

### Cardiovascular disease

There was a significantly higher prevalence of total cardiovascular events in individuals with depression compared to those without depression: 38% (n = 8) for the depressive group vs 15% (n = 6) for non-depressive group (Chi-square test, p = 0.04) with a stronger association in males compared to females (Chi-square test, p = 0.01). Compared to the non-depressive group, those in the depressive group had a 3.5-fold greater odd of having clinical cardiovascular disease (Table 3). This association persisted following adjustment for UFC and become stronger following adjustment for CRP.

**Table 3:** Relative odds (95% confidence intervals) of clinical cardiovascular disease by depressive status

***Model***	***OR***	***95% CI***	***p-value***
***Unadjusted***	3.49	(1.01, 12.0)	0.048
***UFC***-***adjusted***	3.88	(1.00, 15.0)	0.05
***CRP***-***adjusted***	7.44	(1.65, 33.5)	0.009
***UFC and CRP adjusted***	9.51	(1.75, 51.7)	0.009

Reference group: Non-depressive; UFC = urine free cortisol; CRP = C-reactive protein.

There was a trend toward a higher prevalence of atherosclerotic plaque among depressed compared to non-depressed individuals (75% for the depressive group vs 44% for non-depressive group; p = 0.07); however, the difference was not statistically significant, likely due to the smaller number of patients (n = 37) who underwent carotid ultrasound measurements. There was no significant difference in carotid intimal-medial thickness or ankle-brachial indices between the two groups (data not shown).

## Discussion

Multiple biological links that potentially mediate the adverse effect of comorbid depression on diabetes-related and cardiovascular mortality have been described. These include increased pro-inflammatory cytokines, abnormalities of the hypothalamic pituitary axis (HPA), changes in homeostasis between the sympathetic and parasympathetic nervous systems and changes in metabolism [16-19].

Our study found that individuals with diabetes and depression had significantly higher 24-h urine free cortisol levels and CRP than individuals without depression. While there was a trend toward higher late-evening salivary cortisol in individuals with depression compared to those without depression, this difference was not statistically significant.

There were no differences in glycemic and metabolic control or subclinical atheroscleorsis between the two groups. This association persisted following adjustment for UFC and became stronger after adjusting for CRP.

Our depressed patients tended to be older in age, to have a longer duration of diabetes and a higher percentage were receiving insulin treatment. However, none of these trends were statistically significant.

Our study confirms prior findings that MDD is associated with subclinical hypercortisolism and HPA axis activation and increased levels of some inflammatory markers [20-23] and that these associations are present among depressed individuals with type 2 diabetes.

We hypothesized that these physiological abnormalities associated with MDD might explain the higher prevalence of clinical cardiovascular events that we found in the depressed individuals. Hypercortisolism produces increased visceral adiposity by stimulating increased adipocyte size and number [24] and visceral adiposity is a risk factor for cardiovascular disease [25]. Activation of the HPA axis results in simultaneous activation of the sympathetic nervous system, which in turn stimulates release of IL-6, inducing an inflammatory cascade [16]. Elevated CRP and IL-6 has been shown to predict development of cardiovascular disease [26]. UFC and inflammatory markers could explain in part our observed association; however, our results should be interpreted with caution given the small sample size.

Our study has several strengths. First, our participants had physician-confirmed diagnosis for both depression and diabetes status using standardized assessment methods as opposed to questionnaires or self-report. We were also able to characterize several biological determinants in the association between depression and cardiovascular disease in individuals with diabetes, including measures of HPA axis activity (i.e. 24-hour urine free cortisol and salivary cortisol) and inflammatory markers.

However, our study has some weaknesses that should be kept in mind when interpreting our data. First, our sample size was small, which may have limited our power to examine other associations. Second, our study was cross-sectional, which does not allow us to determine the temporality of association between depression, biological factors, and clinical cardiovascular events among those with diabetes. Finally, our participants were recruited from one race/ethnicity, which may limit the generalizability of findings to other populations.

## Conclusions

Our study showed that people with diabetes and depression are more likely to have cardiovascular events than non-depressed patients. The higher levels of CRP and urinary cortisol did not appear to explain the higher number of cardiovascular events, suggesting that other biological factors, such as alterations in the serotonin system, may also be implicated. Larger studies are needed to elucidate the biological mechanisms linking depression to cardiovascular disease in diabetes to identify targets for future preventive interventions.

## Competing interests

The authors declared that they have no competing interests.

## Authors’ contributions

AA conceived the study, participated in its design and coordination and helped to draft the manuscript. JF and MG conceived the study, and participated in its design and coordination. PFD, PS and AK carried out the immunoassays. IB, WM and AC performed cardiovascular measurements. LC, and CB participated in the design of the study and performed the statistical analysis. MMC, LG, LC, CB, CK and LEL participated in the recruitment of patients. All authors read and approved the final manuscript. SHG helped to draft the manuscript.
